# Dietary quality, insulin resistance, and preserved ratio impaired spirometry in postmenopausal women: a cross-sectional study with exploratory mediation analysis

**DOI:** 10.3389/fnut.2026.1897598

**Published:** 2026-07-27

**Authors:** Ziqian Song, Yuan Liu

**Affiliations:** 1 Department of Epidemiology and Health Statistics, School of Public Health, Cheeloo College of Medicine, Shandong University, Jinan, Shandong, China; 2 Shandong Cancer Hospital and Institute, Shandong First Medical University and Shandong Academy of Medical Sciences, Jinan, China

**Keywords:** dietary inflammatory index, dietary quality, insulin resistance, mediation analysis, postmenopausal women, preserved ratio impaired spirometry, sensitivity analysis

## Abstract

**Background:**

Preserved ratio impaired spirometry (PRISm) is a clinically relevant pulmonary phenotype with metabolic heterogeneity. Whether multidimensional dietary quality is associated with PRISm in postmenopausal women remains unclear.

**Objective:**

To examine associations of dietary quality with PRISm and evaluate the exploratory role of insulin resistance.

**Methods:**

This cross-sectional study included 678 postmenopausal women attending routine health examinations at a single hospital in Taian, China, from June 2024 to December 2025. Dietary quality was assessed using the Healthy Eating Index (HEI), Dietary Inflammatory Index (DII), Composite Dietary Antioxidant Index (CDAI), and Diet Index for Gut Microbiota (DI-GM). Insulin resistance was evaluated using HOMA-IR and METS-IR. PRISm was defined as FEV1 < 80% predicted with FEV1/FVC > =0.70. Multivariable regression, restricted cubic splines, exploratory mediation analysis, false-discovery-rate sensitivity analysis, hierarchical adjustment models, quartile-based analyses, restriction to never-smokers, and additional HOMA-IR-adjusted models were performed.

**Results:**

PRISm was identified in 67 participants (9.88%). Higher HEI, DI-GM, and CDAI were associated with lower odds of PRISm, whereas higher DII was associated with higher odds. HOMA-IR was associated with PRISm continuously and categorically. HOMA-IR statistically explained 7.92–10.69% of diet-PRISm associations, but mediation findings did not remain significant after false-discovery-rate correction and should be considered exploratory. Diet-PRISm associations were consistent across hierarchical models and among never-smokers and remained statistically significant after additional adjustment for HOMA-IR. Quartile analyses supported graded associations for HEI, DII, and DI-GM, whereas the categorical CDAI trend was less precise.

**Conclusion:**

In this single-center cross-sectional study, dietary quality and insulin resistance were associated with PRISm in postmenopausal women. The associations were robust across complementary sensitivity analyses; however, the findings remain hypothesis-generating and require longitudinal and multicenter validation.

## 1 Introduction

Preserved ratio impaired spirometry (PRISm) is defined by a reduced forced expiratory volume in one second (FEV1) in the presence of a preserved FEV1/forced vital capacity (FVC) ratio, with interpretation dependent on standardized predicted spirometric values (1). Although this pattern does not meet the conventional spirometric definition of airflow obstruction, it is not a benign finding. Cohort studies and systematic reviews have linked PRISm to impaired respiratory reserve, cardiometabolic comorbidity, cardiovascular events, and excess mortality (2–8). These observations have shifted PRISm from a descriptive spirometric category toward a clinically meaningful phenotype that may integrate pulmonary, metabolic, and inflammatory pathways.

The metabolic dimension of PRISm is particularly important in women after menopause. The menopausal transition is accompanied by endocrine changes that favor visceral adiposity, dyslipidemia, deterioration of insulin sensitivity, and low-grade systemic inflammation (9–14). These changes may intensify the pulmonary consequences of poor diet, oxidative stress, and glucose-metabolic disturbance. In parallel, epidemiologic and longitudinal evidence indicates that insulin resistance, diabetes-related metabolic dysfunction, and reduced ventilatory capacity are interconnected, supporting a plausible metabolic-pulmonary axis in postmenopausal women (15–19).

Dietary quality is a modifiable determinant of metabolic homeostasis. Healthier dietary patterns rich in plant foods, fiber, micronutrients, and antioxidant components are associated with improved insulin sensitivity and lower cardiometabolic risk, whereas pro-inflammatory dietary patterns are linked to obesity, insulin resistance, and chronic inflammation (20–23). The Healthy Eating Index (HEI), Dietary Inflammatory Index (DII), Diet Index for Gut Microbiota (DI-GM), and Composite Dietary Antioxidant Index (CDAI) capture distinct but complementary dimensions of diet quality, inflammatory potential, microbiota-related dietary features, and antioxidant capacity (24–27).

Despite the biological plausibility of a diet-metabolism-lung function axis, evidence focusing on postmenopausal women in real-world clinical settings remains limited. Moreover, the robustness of these associations across alternative model specifications and clinically relevant subgroups has not been adequately characterized. We therefore analyzed a hospital-based health-examination dataset using multivariable regression, restricted cubic splines, exploratory mediation analysis, and complementary sensitivity analyses. This analytic structure was designed to combine etiologic interpretability with assessment of dose–response patterns and robustness while maintaining cautious interpretation of cross-sectional mediation.

## 2 Materials and methods

### 2.1 Study design and participants

This was a cross-sectional analysis of a single-center hospital-based health-examination dataset. Participants were women who voluntarily attended routine health examinations at the Health Examination Center of the Second Affiliated Hospital of Shandong First Medical University, Taian, China, between June 2024 and December 2025. They were not recruited because of a specific respiratory disease diagnosis. The analytic dataset contained 678 postmenopausal women with complete information on spirometric status, dietary indices, insulin resistance indices, demographic characteristics, lifestyle factors, hypertension status, total energy intake, and age at menopause.

Postmenopausal status was defined according to clinical history. From 8,346 screened health-examination attendees, 1,658 were identified as postmenopausal women. We then sequentially excluded women with incomplete dietary data (*n* = 633), incomplete routine blood examination data (*n* = 47), previous physician-diagnosed pulmonary disease (*n* = 91), incomplete spirometric examination (*n* = 135), and incomplete other covariate data (*n* = 74), leaving a final complete-case analytic sample of 678 participants.

### 2.2 Pulmonary outcome

Spirometric parameters were obtained during routine health examination. PRISm was defined as FEV1 < 80% of the predicted value with preserved FEV1/FVC ratio > = 0.70. Participants not meeting this definition were classified as having non-PRISm spirometry. Predicted values were calculated using the GLI 2012 reference equations, and because all participants were Chinese, race was uniformly entered as the ‘Other’ category in the clinical spirometry workflow.

### 2.3 Dietary exposures

Dietary intake was collected on the day of the health examination using a self-reported 24-h dietary recall referring to food and beverage intake on the preceding day. Data collection did not involve a trained interviewer or dietitian. Total energy intake was derived from the same self-reported recall data. Four dietary indices were then calculated as complementary exposures: HEI reflected overall dietary quality; DII quantified the inflammatory potential of the diet; DI-GM reflected dietary characteristics linked to a favorable gut microbiota profile; and CDAI represented the combined antioxidant capacity of dietary intake (24–27). In regression models, HEI was scaled per 10-point increment to improve interpretability, whereas DII, DI-GM, and CDAI were analyzed per 1-unit increment. Because single-day self-reported recall may not represent usual long-term diet and may introduce recall and reporting error, the dietary assessment method was treated as an important limitation.

### 2.4 Insulin resistance indices

Insulin resistance was assessed using HOMA-IR and METS-IR. HOMA-IR was calculated from fasting insulin and fasting glucose (28). METS-IR was calculated from fasting glucose, triglycerides, body mass index, and high-density lipoprotein cholesterol according to the established formula (29). HOMA-IR and METS-IR were modeled as continuous variables; dichotomous analyses used HOMA-IR > =2.80 and METS-IR > =32.80 as elevated insulin resistance thresholds (30, 31).

### 2.5 Covariates

Covariates were selected *a priori* according to biological plausibility and availability in the hospital dataset. Fully adjusted models included age, education level, socioeconomic status, marital status, total energy intake, smoking behavior, alcohol consumption, hypertension, and age at menopause. Education was categorized as less than high school, high school, or more than high school. Socioeconomic status was recorded as low, middle, or high according to a composite hospital registration assessment incorporating household income, education/occupation-related information, medical insurance, and residential information. Smoking behavior was categorized as never, former, or current smoking. Alcohol consumption was categorized as never, former, mild, moderate, or heavy consumption in descriptive analyses; for multivariable modeling, moderate and heavy alcohol consumption were combined because the heavy-drinking group was very small and contained no PRISm cases. Hypertension was recorded as yes or no. Physical activity, individual BMI, waist circumference, diabetes diagnosis, glucose-lowering medication use, inflammatory biomarkers, microbiome data, and other medication variables were not available in the analytic dataset and therefore could not be included as covariates.

### 2.6 Statistical analysis

Continuous variables are reported as mean (standard deviation), and categorical variables as count (percentage). Between-group differences according to PRISm status were evaluated using Welch t tests for continuous variables and chi-square tests for categorical variables. These descriptive tests were not used as covariate-selection procedures. Multivariable models were adjusted for age, education level, socioeconomic status, marital status, total energy intake, smoking behavior, alcohol consumption, hypertension, and age at menopause.

Associations between dietary indices and PRISm were estimated using logistic regression. Associations between dietary indices and insulin resistance were estimated using robust linear regression with the Huber M-estimator to reduce sensitivity to non-normality and influential observations. Associations between insulin resistance indices and PRISm were evaluated using logistic regression. Restricted cubic spline models with three knots placed at the 10th, 50th, and 90th empirical percentiles were used to assess dose–response patterns and non-linearity. Overall associations were assessed using likelihood-ratio tests comparing each full spline model with the corresponding covariate-only model, whereas non-linearity was assessed by comparing the spline model with the corresponding linear-exposure model.

Exploratory mediation analyses were performed to decompose the diet-PRISm association into total, direct, and indirect components through HOMA-IR or METS-IR. Because the outcome was binary and the analysis was cross-sectional, mediation was interpreted as a statistical decomposition rather than causal proof. Indirect effects and proportions mediated were estimated using a linear-probability decomposition with 1,000 bootstrap resamples. This approach was chosen for transparent risk-difference-scale decomposition, but it is not a counterfactual causal mediation analysis and may produce predicted probabilities outside the 0–1 range. Point estimates and percentile bootstrap 95% confidence intervals were reported for total effects, direct effects, indirect effects, and proportions mediated. Because eight mediation pathways were examined, mediation findings were interpreted cautiously and were considered exploratory (32).

Additional sensitivity analyses were conducted to evaluate the robustness of the diet-PRISm associations. First, associations were estimated in unadjusted, minimally adjusted, and fully adjusted logistic regression models. The minimally adjusted model included age, total energy intake, and age at menopause; the fully adjusted model included age, education level, socioeconomic status, marital status, total energy intake, smoking behavior, alcohol consumption, hypertension, and age at menopause. Second, each dietary index was categorized using empirical quartile cut points, with the lowest quartile as the reference. *p* values for trend were calculated by assigning the category-specific median to each participant and modeling this value continuously. Because no PRISm cases occurred in the highest DI-GM quartile, the third and fourth DI-GM quartiles were combined to obtain stable estimates. Third, the fully adjusted analyses were repeated among never-smokers. Fourth, HOMA-IR was added to the fully adjusted diet-PRISm models to assess whether the associations persisted after accounting for insulin resistance. Because HOMA-IR may lie on the hypothesized pathway, this last analysis was interpreted as an attenuation analysis rather than as a causal direct-effect estimate.

Benjamini–Hochberg false-discovery-rate adjustment was performed as sensitivity analyses separately for the 16 principal regression tests and for the eight mediation indirect-effect tests. Analyses were conducted using Python 3.13.5, numpy 2.3.5, pandas 2.2.3, scipy 1.17.0, and statsmodels 0.14.6. A two-sided *p* value <0.05 was considered statistically significant, with emphasis placed on effect sizes, confidence intervals, consistency across sensitivity analyses, and the exploratory nature of multiple comparisons.

## 3 Results

### 3.1 Baseline characteristics

The final hospital-based analytic dataset included 678 postmenopausal women. (Table 1) PRISm was present in 67 participants, corresponding to a prevalence of 9.88%. Compared with women without PRISm, those with PRISm were older, had a higher prevalence of hypertension, and had a later age at menopause. They also showed less favorable dietary and metabolic profiles, including lower HEI, CDAI, and DI-GM, higher DII, and higher HOMA-IR and METS-IR values. Education, marital status, socioeconomic status, smoking behavior, and alcohol consumption did not differ significantly by PRISm status. Because the sample consisted of voluntary single-center health-examination attendees, this prevalence should not be interpreted as a population-representative estimate.

**Table 1:** Baseline characteristics of the postmenopausal women according to PRISM status.

Variable	PRISm			
	Total	No	Yes	*p*-value
Age, years	58.73 (6.72)	58.52 (6.65)	60.60 (7.08)	0.03
Total energy intake, kcal/day	1646.92 (338.99)	1655.73 (332.37)	1566.67 (387.98)	0.08
Age at menopause, years	49.19 (4.11)	49.05 (4.16)	50.46 (3.47)	<0.01
HEI	52.29 (11.24)	52.75 (11.09)	48.15 (11.83)	<0.01
DII	1.70 (1.33)	1.64 (1.30)	2.28 (1.48)	<0.01
CDAI	0.11 (2.79)	0.21 (2.76)	−0.78 (2.86)	0.01
DI-GM	4.89 (1.02)	4.93 (1.03)	4.54 (0.82)	<0.01
HOMA-IR	2.48 (1.09)	2.43 (1.08)	2.92 (1.09)	<0.01
METS-IR	30.91 (4.94)	30.74 (4.92)	32.52 (4.87)	0.01
Education level				0.64
More than high school	322 (47.49%)	291 (47.63%)	31 (46.27%)	
High school	232 (34.22%)	211 (34.53%)	21 (31.34%)	
Less than high school	124 (18.29%)	109 (17.84%)	15 (22.39%)	
Socioeconomic status				0.47
High	191 (28.17%)	171 (27.99%)	20 (29.85%)	
Middle	347 (51.18%)	310 (50.74%)	37 (55.22%)	
Low	140 (20.65%)	130 (21.28%)	10 (14.93%)	
Marital status				0.56
Married/living with partner	495 (73.01%)	448 (73.32%)	47 (70.15%)	
Widowed/divorced/separated	152 (22.42%)	134 (21.93%)	18 (26.87%)	
Never married	31 (4.57%)	29 (4.75%)	2 (2.99%)	
Smoking behavior				0.40
Never	548 (80.83%)	498 (81.51%)	50 (74.63%)	
Former	92 (13.57%)	80 (13.09%)	12 (17.91%)	
Now	38 (5.60%)	33 (5.40%)	5 (7.46%)	
Alcohol consumption				0.53
Never	466 (68.73%)	425 (69.56%)	41 (61.19%)	
Former	44 (6.49%)	39 (6.38%)	5 (7.46%)	
Mild	112 (16.52%)	98 (16.04%)	14 (20.90%)	
Moderate	50 (7.37%)	43 (7.04%)	7 (10.45%)	
Heavy	6 (0.88%)	6 (0.98%)	0 (0.00%)	
Hypertension				0.02
No	392 (57.82%)	363 (59.41%)	29 (43.28%)	
Yes	286 (42.18%)	248 (40.59%)	38 (56.72%)	

Values are mean (standard deviation) for continuous variables or *n* (%) for categorical variables.

### 3.2 Dietary indices, insulin resistance, and PRISm

In fully adjusted logistic regression models, healthier dietary indices were inversely associated with PRISm. (Table 2) Each 10-point increase in HEI was associated with 34.00% lower odds of PRISm (OR = 0.66, 95% CI: 0.52–0.84), and each 1-point increase in DI-GM was associated with 34.00% lower odds (OR = 0.66, 95% CI: 0.51–0.87). CDAI also showed an inverse association (OR = 0.90, 95% CI: 0.81–0.98). In contrast, DII was positively associated with PRISm (OR = 1.44, 95% CI: 1.18–1.76), suggesting that a more pro-inflammatory diet was related to greater likelihood of this spirometric phenotype.

**Table 2:** Fully adjusted associations of dietary indices with PRISm and insulin resistance.

Dietary index	PRISm		HOMA-IR		METS-IR	
	OR (95% CI)	*p*-value	*β* (95% CI)	*p*-value	*β* (95% CI)	*p*-value
HEI per 10-point increase	0.66 (0.52, 0.84)	<0.01	−0.15 (−0.22, −0.07)	<0.01	−0.85 (−1.18, −0.51)	<0.01
DII per 1-unit increase	1.44 (1.18, 1.76)	<0.01	0.18 (0.11, 0.24)	<0.01	0.52 (0.23, 0.80)	<0.01
DI-GM per 1-point increase	0.66 (0.51, 0.87)	<0.01	−0.12 (−0.20, −0.04)	<0.01	−0.34 (−0.71, 0.03)	0.07
CDAI per 1-unit increase	0.90 (0.81, 0.98)	0.02	−0.03 (−0.06, 0.00)	0.05	−0.20 (−0.34, −0.06)	<0.01

Model adjusted for age, education level, socioeconomic status, marital status, total energy intake, smoking behavior, alcohol consumption, hypertension, and age at menopause. HEI was scaled per 10-point increase; other dietary indices were scaled per 1-unit increase. Linear regression coefficients describe absolute change in insulin resistance indices. In multivariable models, moderate and heavy alcohol consumption were combined because the heavy-drinking group was sparse.

Dietary indices were also associated with insulin resistance. Higher HEI was associated with lower HOMA-IR and METS-IR, whereas higher DII was associated with higher values of both indices. DI-GM showed a significant inverse association with HOMA-IR and a weaker, non-significant association with METS-IR after full adjustment. CDAI was inversely associated with METS-IR, while its association with HOMA-IR was borderline. These findings support a metabolic gradient linking diet quality with insulin resistance, while also showing that HOMA-IR and METS-IR may capture related but non-identical metabolic information.

### 3.3 Insulin resistance and PRISm

Insulin resistance was consistently associated with PRISm. (Table 3) In the fully adjusted model, each 1-unit increase in HOMA-IR was associated with higher odds of PRISm (OR = 1.41, 95% CI: 1.10–1.81). Participants with HOMA-IR > =2.80 had more than threefold higher odds of PRISm (OR = 3.32, 95% CI: 1.90–5.81). METS-IR modeled per 5-unit increase was also associated with PRISm (OR = 1.36, 95% CI: 1.03–1.79), whereas the dichotomous METS-IR threshold was not statistically significant.

**Table 3:** Fully adjusted associations between insulin resistance indices and PRISm.

Insulin resistance index	OR (95% CI)	*p*-value
HOMA-IR per 1-unit increase	1.41 (1.10, 1.81)	0.01
HOMA-IR > =2.80		
No		
Yes	3.32 (1.90, 5.81)	<0.01
METS-IR per 5-unit increase	1.36 (1.03, 1.79)	0.03
METS-IR > =32.80		
No		
Yes	1.38 (0.80, 2.37)	0.24

Model adjusted for age, education level, socioeconomic status, marital status, total energy intake, smoking behavior, alcohol consumption, hypertension, and age at menopause. In multivariable models, moderate and heavy alcohol consumption were combined because the heavy-drinking group was sparse.

In Benjamini–Hochberg false-discovery-rate sensitivity analyses of the 16 principal regression tests, all nominally significant principal regression associations retained q < 0.05. The associations of DI-GM with METS-IR, CDAI with HOMA-IR, and dichotomous METS-IR with PRISm were not FDR-significant, consistent with their non-significant or borderline nominal *p* values.

### 3.4 Dose–response analyses

**Figure 1:**
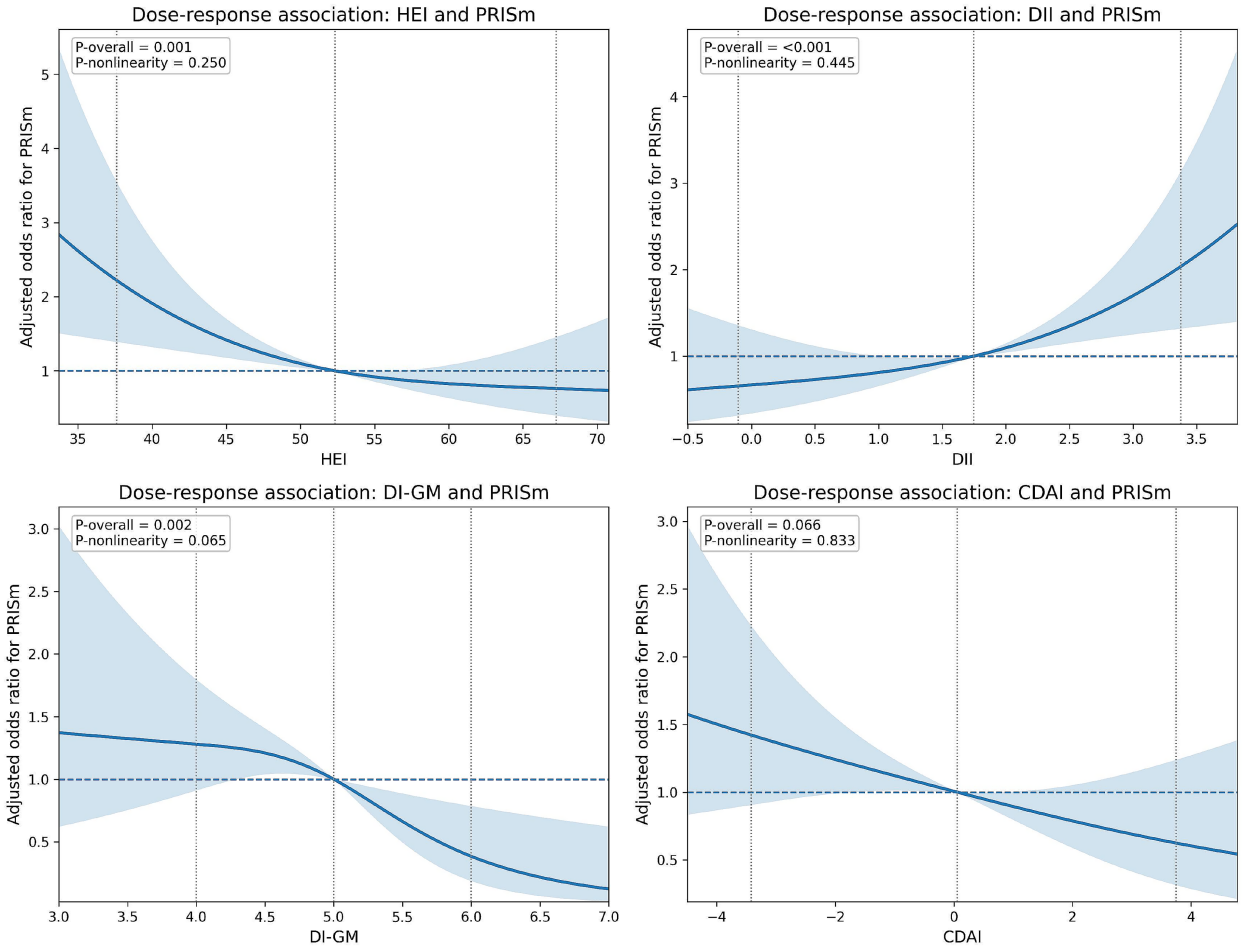
Adjusted dose–response curves for dietary indices and PRISm from three-knot restricted cubic spline models. Knots were placed at the 10th, 50th, and 90th empirical percentiles and are shown by vertical dotted lines. Odds ratios were estimated from fully adjusted models and centered at the median of each dietary index. Shaded bands indicate 95% confidence intervals. Overall and non-linearity P values were obtained from likelihood-ratio tests.

Three-knot restricted cubic spline analyses showed significant overall associations of HEI (*p* = 0.001), DII (*p* < 0.001), and DI-GM (*p* = 0.002) with PRISm, whereas CDAI showed a borderline but non-significant overall association (*p* = 0.066). No dietary index showed statistically significant evidence of non-linearity: the *p* values for non-linearity were 0.250 for HEI, 0.445 for DII, 0.065 for DI-GM, and 0.833 for CDAI. Although the DI-GM curve suggested a possible departure from linearity, the statistical evidence was borderline and was not interpreted as definitive. The revised spline curves are shown in Figure 1, and the overall and non-linearity tests together with the exact three-knot locations are reported in Table 4.

**Table 4:** Three-knot restricted cubic spline tests for dietary indices and PRISM.

Variable	*p* for overall	*p* for non-linearity
HEI	0.001	0.250
DII	<0.001	0.445
DI-GM	0.002	0.065
CDAI	0.066	0.833

Overall *p*-values test the dietary exposure jointly by comparing the full spline model with the covariate-only model; non-linearity p values compare the spline model with the corresponding linear exposure model. Models were adjusted for the full covariate set. Three knots were placed at the 10th, 50th, and 90th empirical percentiles. The knot values were as follows: HEI (per 10 points), 3.76, 5.23, and 6.72; DII, −0.10, 1.75, and 3.37; DI-GM, 4.00, 5.00, and 6.00; and CDAI, −3.42, 0.05, and 3.75. Figure 1 was generated using these same knot locations and was centered at the median of each dietary index.

### 3.5 Exploratory mediation by insulin resistance

Exploratory mediation analysis suggested that HOMA-IR explained a modest but consistent portion of the associations between dietary indices and PRISm. (Table 5) The estimated proportions mediated through HOMA-IR were 9.99% for HEI, 10.69% for DII, 8.47% for DI-GM, and 7.92% for CDAI. Bootstrap confidence intervals for the HOMA-IR indirect effects did not cross zero for these four pathways; however, after Benjamini–Hochberg correction across the eight mediation indirect-effect tests, none of the indirect effects remained significant at q < 0.05. In contrast, mediation through METS-IR was weaker and the bootstrap confidence intervals for the indirect effects included zero. These results suggest that fasting insulin-based insulin resistance may better capture the metabolic pathway connecting diet to PRISm in this dataset; however, the mediated proportions were small, indicating that most of the diet-PRISm association likely operates through other pathways or through residual confounding.

**Table 5:** Exploratory mediation of dietary index-PRISm associations by HOMA-IR and METS-IR.

Exposure	Mediator	Total effect (95% CI)	Direct effect (95% CI)	Indirect effect (95% CI)	Proportion mediated, % (95% CI)	*p* for indirect effect
HEI per 10-point increase	HOMA-IR	−0.03 (−0.06, −0.01)	−0.03 (−0.05, −0.01)	−3.46E-03 (−7.69E-03, −5.10E-04)	9.99 (1.91, 24.87)	0.03
	METS-IR	−0.03 (−0.06, −0.01)	−0.03 (−0.05, −0.01)	−3.25E-03 (−7.84E-03, 1.26E-03)	9.38 (−4.38, 31.96)	0.16
DII per 1-unit increase	HOMA-IR	0.03 (0.01, 0.05)	0.03 (0.01, 0.05)	3.42E-03 (2.20E-04, 7.49E-03)	10.69 (0.82, 29.34)	0.03
	METS-IR	0.03 (0.01, 0.05)	0.03 (0.01, 0.05)	2.05E-03 (−5.80E-04, 5.06E-03)	6.42 (−2.32, 19.14)	0.12
DI-GM per 1-point increase	HOMA-IR	−0.03 (−0.05, −0.01)	−0.03 (−0.05, −0.01)	−2.83E-03 (−6.82E-03, −3.00E-04)	8.47 (0.93, 24.36)	0.02
	METS-IR	−0.03 (−0.05, −0.02)	−0.03 (−0.05, −0.01)	−1.38E-03 (−4.61E-03, 3.50E-04)	4.14 (−1.21, 14.51)	0.14
CDAI per 1-unit increase	HOMA-IR	−9.92E-03 (−1.81E-02, −2.43E-03)	−9.14E-03 (−1.71E-02, −1.85E-03)	−7.90E-04 (−1.92E-03, −4.00E-05)	7.92 (0.27, 32.00)	0.03
	METS-IR	−9.92E-03 (−1.78E-02, −2.25E-03)	−9.06E-03 (−1.73E-02, −5.70E-04)	−8.70E-04 (−2.25E-03, 6.00E-05)	8.73 (−0.96, 58.17)	0.08

Total, direct, and indirect effects were estimated using linear-probability decomposition with 1,000 bootstrap resamples. Values in parentheses are percentile bootstrap 95% confidence intervals. Because the design was cross-sectional, mediation should be interpreted as exploratory and not causal.

### 3.6 Sensitivity analyses

The continuous diet-PRISm associations were stable across unadjusted, minimally adjusted, and fully adjusted models (Table 6). Additional adjustment for HOMA-IR modestly attenuated the estimates, but all four associations remained statistically significant: HEI, OR = 0.69 per 10-point increase (95% CI: 0.54–0.88; *p* = 0.003); DII, OR = 1.38 per 1-unit increase (95% CI: 1.12–1.69; *p* = 0.002); DI-GM, OR = 0.69 per 1-point increase (95% CI: 0.53–0.90; *p* = 0.006); and CDAI, OR = 0.91 per 1-unit increase (95% CI: 0.82–1.00; *p* = 0.043). Analyses restricted to never-smokers included 548 women and 50 PRISm cases and produced directionally consistent, statistically significant estimates for all four dietary indices.

**Table 6:** Sensitivity analyses of continuous dietary indices in relation to PRISm.

Dietary index		Unadjusted	Minimally adjusted	Fully adjusted	Fully adjusted + HOMA-IR	Never-smokers
HEI per 10-point increase	OR (95% CI)	0.69 (0.55, 0.87)	0.68 (0.54, 0.86)	0.66 (0.52, 0.84)	0.69 (0.54, 0.88)	0.67 (0.51, 0.89)
	*p*-value	0.002	0.001	<0.001	0.003	0.005
DII per 1-unit increase	OR (95% CI)	1.45 (1.19, 1.76)	1.43 (1.18, 1.74)	1.44 (1.18, 1.76)	1.38 (1.12, 1.69)	1.50 (1.18, 1.90)
	*p*-value	<0.001	<0.001	<0.001	0.002	<0.001
DI-GM per 1-point increase	OR (95% CI)	0.68 (0.53, 0.88)	0.70 (0.54, 0.90)	0.66 (0.51, 0.87)	0.69 (0.53, 0.90)	0.67 (0.50, 0.91)
	*p*-value	0.003	0.005	0.003	0.006	0.009
CDAI per 1-unit increase	OR (95% CI)	0.88 (0.80, 0.96)	0.89 (0.81, 0.98)	0.90 (0.81, 0.98)	0.91 (0.82, 1.00)	0.87 (0.78, 0.97)
	*p*-value	0.006	0.014	0.021	0.043	0.014

Values are odds ratios (95% confidence intervals) with two-sided *p*-values. The minimally adjusted model included age, total energy intake, and age at menopause. The fully adjusted model additionally included education level, socioeconomic status, marital status, smoking behavior, alcohol consumption, and hypertension. The HOMA-IR model added HOMA-IR to the fully adjusted covariate set and was interpreted as an attenuation analysis. The never-smoker analysis included 548 participants and 50 PRISm cases and omitted smoking behavior from the covariate set. Moderate and heavy alcohol consumption were combined in multivariable models.

Quartile-based analyses further supported graded associations for HEI, DII, and DI-GM (Table 7). Compared with the lowest quartile, the highest HEI quartile was associated with lower odds of PRISm (OR = 0.28, 95% CI: 0.12–0.62; P for trend <0.001), whereas the highest DII quartile was associated with higher odds (OR = 2.97, 95% CI: 1.39–6.34; P for trend = 0.001). For DI-GM, the third and fourth quartiles were combined because the highest quartile contained no PRISm cases; the combined upper category was associated with lower odds relative to the lowest category (OR = 0.27, 95% CI: 0.12–0.61; P for trend = 0.001). CDAI estimates in the upper quartiles were directionally inverse but less precise (P for trend = 0.078).

**Table 7:** Quartile-based associations of dietary indices with PRISm.

Dietary index/category	Participants/PRISm cases	Adjusted OR (95% CI)	*p*-value	*p* for trend
HEI Q1 (<=44.93)	170/27	Reference		
HEI Q2 (>44.93–52.32)	169/17	0.55 (0.28, 1.09)	0.088	
HEI Q3 (>52.32–59.90)	169/14	0.40 (0.20, 0.82)	0.012	
HEI Q4 (>59.90)	170/9	0.28 (0.12, 0.62)	0.002	<0.001
DII Q1 (<=0.75)	170/11	Reference	–	
DII Q2 (>0.75–1.75)	169/11	1.04 (0.43, 2.51)	0.935	
DII Q3 (>1.75–2.63)	169/17	1.91 (0.84, 4.31)	0.122	
DII Q4 (>2.63)	170/28	2.97 (1.39, 6.34)	0.005	0.001
DI-GM Q1 (<=4)	236/33	Reference		
DI-GM Q2 (=5)	264/26	0.59 (0.34, 1.05)	0.074	
DI-GM Q3-Q4 (> = 6)	178/8	0.27 (0.12, 0.61)	0.002	0.001
CDAI Q1 (<= − 1.75)	170/21	Reference		
CDAI Q2 (> − 1.75–0.05)	169/22	1.14 (0.59, 2.23)	0.692	
CDAI Q3 (>0.05–1.86)	169/12	0.56 (0.26, 1.21)	0.141	
CDAI Q4 (>1.86)	170/12	0.59 (0.27, 1.28)	0.185	0.078

Quartile cut points were empirical. Models were adjusted for age, education level, socioeconomic status, marital status, total energy intake, smoking behavior, alcohol consumption, hypertension, and age at menopause. *p* for trend used category-specific medians. Because no PRISm cases occurred in the highest DI-GM quartile, Q3 and Q4 were combined. OR, odds ratio; CI, confidence interval.

## 4 Discussion

In this hospital-based study of postmenopausal women, multidimensional dietary quality was associated with PRISm. Higher HEI, DI-GM, and CDAI were associated with lower odds of PRISm, whereas higher DII was associated with higher odds. These findings are consistent with prior evidence that PRISm is clinically heterogeneous and metabolically enriched, and they further suggest that diet-related metabolic disturbance may be relevant in a voluntary hospital health-examination population (2–8, 15–19).

The observed PRISm prevalence of 9.88% should be interpreted in light of the single-center health-examination sampling frame. Population-based or large Asian datasets have reported different estimates, including a 24.8% age, sex, and region-standardized prevalence among Chinese adults aged 40 years or older in the China Kadoorie Biobank, an average prevalence of 10.4% in the Korean KNHANES 2010–2019 survey, and a 16.7% prevalence in the Japanese OCEAN health-examination study (33–35). Differences in age structure, sex distribution, smoking, obesity, comorbidity, recruitment setting, spirometry workflow, and reference equations may partly explain why our estimate is lower than some population-based Asian estimates. In addition, voluntary health-examination attendees may be healthier, more health-conscious, and of higher socioeconomic status than the general postmenopausal population, which could shift dietary-index distributions and attenuate or distort prevalence estimates.

The positive association between DII and PRISm is biologically plausible. A pro-inflammatory dietary profile may amplify systemic low-grade inflammation, oxidative stress, endothelial dysfunction, and adipose-tissue inflammatory signaling, all of which may influence airway remodeling, pulmonary microvascular function, and mechanical ventilatory limitation. Prior nutritional epidemiology links antioxidant intake, fiber intake, and dietary pattern quality with lung function or chronic respiratory disease, while gut-lung axis research supports biologic crosstalk between diet, microbial metabolites, immune regulation, and respiratory health (36–45).

Insulin resistance emerged as a key metabolic correlate of PRISm. HOMA-IR showed robust associations with PRISm both as a continuous measure and using a clinically meaningful threshold. METS-IR also captured a significant continuous gradient, although its categorical threshold was less stable. These observations align with evidence that impaired glucose metabolism, adiposity-related mechanical loading, and insulin-resistance signaling can adversely affect ventilatory capacity, pulmonary inflammation, and structural remodeling (46–48).

The reanalyzed three-knot dose–response models added nuance to the regression findings. HEI and DII showed significant overall associations with PRISm without evidence of non-linear departures, supporting broadly graded relationships rather than abrupt threshold effects. DI-GM retained a significant overall association, but its non-linearity test was borderline; therefore, the apparent curvature should be regarded as suggestive rather than definitive. CDAI did not show a statistically significant overall spline association, although the fully adjusted linear logistic regression model showed an inverse association with PRISm. This distinction highlights the importance of interpreting the dose–response shape separately from the linear trend and of avoiding overstatement of knot-sensitive non-linearity.

The newly added sensitivity analyses strengthen the internal robustness of the epidemiologic findings. Similar estimates across progressive levels of adjustment and in the restriction to never-smokers indicate that the observed associations were not explained solely by basic demographic factors, energy intake, or smoking status. Associations also persisted after additional adjustment for HOMA-IR, suggesting that insulin resistance did not fully account for the diet-PRISm relationships in this dataset. However, because HOMA-IR may be an intermediate variable, these attenuation models should not be interpreted as identifying causal direct effects. The quartile analyses corroborated graded patterns for HEI, DII, and DI-GM, whereas the CDAI trend was less precise, which may reflect loss of information and statistical power after categorization. The absence of PRISm cases in the highest DI-GM quartile required combination of the two upper categories and further underscores the sparse-data limitations of categorical analyses.

The findings are particularly relevant to postmenopausal women. Menopause-related changes in body composition, estrogen signaling, adipose distribution, insulin sensitivity, and inflammatory tone may increase susceptibility to diet-related pulmonary impairment. Obesity-related respiratory mechanics and adipose-tissue inflammation may further amplify the association between insulin resistance and impaired spirometric reserve in this life stage (9–14, 46–48).

This study has several strengths. First, it focuses on a clinically relevant and understudied population: postmenopausal women undergoing routine hospital health examination. Second, it evaluates four dietary indices representing different nutritional constructs rather than relying on a single diet score. Third, it integrates conventional regression, non-linear modeling, exploratory mediation, multiple-testing sensitivity analysis, hierarchical adjustment, quartile-based analyses, restriction to never-smokers, and HOMA-IR-adjusted attenuation models. Fourth, the revised Methods section provides additional information on study site, enrollment period, self-referred health-examination recruitment, dietary assessment, socioeconomic-status definition, spirometric reference equations, software, and the rationale and implementation of the sensitivity analyses.

Several limitations should also be acknowledged. The cross-sectional design precludes causal inference, and reverse causation cannot be excluded. Women with subclinical respiratory impairment may have modified their diet, reduced physical activity, changed body weight, or sought medical advice before the health examination; these changes could secondarily influence dietary scores and insulin resistance, making the observed diet-insulin resistance-PRISm pattern non-directional. The mediation analysis therefore cannot establish a temporal sequence from diet to insulin resistance to PRISm and should be interpreted only as exploratory statistical decomposition.

The single-center, voluntary health-examination design may introduce healthy-volunteer and selection biases. Individuals who attend routine check-ups may differ from the broader postmenopausal population in health awareness, socioeconomic status, comorbidity burden, diet, and access to preventive care. This selection mechanism may affect both the dietary-index distribution and the PRISm prevalence estimate, limiting generalizability. Dietary data were derived from a self-reported 24-h recall of the preceding day’s intake rather than repeated recalls or a validated long-term food-frequency questionnaire, and thus may be affected by random day-to-day variation, reporting error, and the lack of interviewer- or dietitian-assisted probing. Detailed spirometry quality-control records, ATS/ERS acceptability and repeatability indicators, calibration logs, and operator reliability measures were not retained in the analytic dataset, which may affect confidence in PRISm classification. In addition, physical activity, individual BMI, waist circumference, diabetes diagnosis, glucose-lowering medication use, inflammatory biomarkers, microbiome data, and other medication variables were unavailable, leaving potential residual confounding. The BMI issue is especially important: BMI is part of the pre-computed METS-IR score, but the individual BMI variable was not available for separate adjustment. Adjusting for BMI in METS-IR models could also create over-adjustment because BMI is a component of the exposure; therefore, we reported METS-IR models without additional BMI adjustment and explicitly recognize the remaining analytical tension. Finally, the complete-case analytic file did not allow formal evaluation of missing-data mechanisms, and the limited number of PRISm cases reduced precision, particularly in categorical and subgroup analyses. Future prospective, multicenter studies with repeated dietary assessment, standardized spirometry quality metrics, lung-volume testing, physical activity, adiposity measures, medication data, inflammatory biomarkers, microbiome data, and larger independent cohorts are needed.

## 5 Conclusion

Among postmenopausal women in a single-center hospital-based health-examination dataset, better overall dietary quality, lower dietary inflammatory potential, higher antioxidant capacity, and more favorable gut microbiota-related dietary characteristics were associated with lower odds of PRISm. Insulin resistance, particularly HOMA-IR, was strongly associated with PRISm and statistically explained a small proportion of the diet-PRISm associations. The diet-PRISm associations were consistent across complementary sensitivity analyses, including progressive covariate adjustment, quartile-based modeling, restriction to never-smokers, and additional adjustment for HOMA-IR. These findings support dietary quality and metabolic health as potentially relevant dimensions of PRISm evaluation in postmenopausal women, while the cross-sectional design, residual confounding, and limited event count require cautious interpretation and prospective confirmation.

## Data Availability

The original contributions presented in the study are included in the article/supplementary material, further inquiries can be directed to the corresponding author.
